# BIA400 versus Ponto Wide: a long-term retrospective analysis of implant stability, tolerability and survival in bone conduction devices

**DOI:** 10.1017/S0022215126105027

**Published:** 2026-08

**Authors:** Florine de Haan, Jeroen Peters, Pauline van Kempen, Ruben Strijbos, Steven Bom

**Affiliations:** Otorhinolaryngology, Deventer Hospital, Netherlands

**Keywords:** BAHA, hearing aids, implantable hearing aids, osseo-integrated implants, statistics

## Abstract

**Objective:**

The purpose of this single-centre retrospective study was to determine the long-term outcomes of the BIA400 and Ponto Wide, in terms of implant stability, soft tissue reactions, skin thickening and implant loss.

**Methods:**

Adults who had the BIA400 or Ponto Wide implanted between January 2013 and December 2023 and had at least two follow-up appointments were included, leading to a cohort of 93 BIA400 and 114 Ponto Wide implants.

**Results:**

Significantly more soft tissue reactions with a Holgers Scale score of 1 or greater (*p* = 0.003), adverse soft tissue reactions of Holgers Scale score of 2 or greater (*p* = 0.018), skin thickening (*p* < 0.001) and skin thickening requiring treatment (*p* ≤ 0.001) occurred in the BIA400 group. No difference in implant stability was found. One BIA400 implant (1.1 per cent) and three Ponto Wide implants (2.7 per cent) were lost.

**Conclusion:**

Both implants have excellent implant stability and similar implant survival rates. The Ponto Wide has favourable outcomes in terms of (adverse) soft tissue reaction and skin thickening (requiring treatment).

## Introduction

Bone conduction devices (BCDs) can offer an implantable solution for patients with conductive and mixed hearing loss or sensorineural hearing loss with either single sided deafness or chronic otitis externa, for whom conventional hearing aids were not an option.^1^ Tjellström and Håkansson, founders of the BCD,^2^ marketed their product in collaboration with the ancestor of Cochlear under the brand name Baha: Bone-Anchored Hearing Aid. More recently, a second manufacturer, Oticon Medical, introduced a BCD, namely the Ponto-system.

Since May 2014, only the newest designs from Cochlear and Oticon, the BIA400 and Ponto Wide, respectively, have been implanted in Deventer Hospital. The BIA400 is the successor of the BIA300, and is the combination of the BI300 implant and BA400 abutment. The coating promotes the integration of soft tissue by allowing more cell binding proteins to bind to the surface.^3^^,^^4^ The abutments are available in sizes of 6, 8, 10 and 12 mm. The Ponto Wide is a titanium implant from Oticon and is combined with a smooth surface titanium abutment. Compared to Cochlear’s and Oticon’s previous designs, the flange fixture and Ponto regular, respectively, these implants have a wider diameter (4.5 mm instead of 3.75 mm) which is known to be an important factor in implant stability.^5^

Currently, solely based on the patient’s preferences for sound transducer, the corresponding implant is chosen. After all, not every sound transducer is compatible with every implant. However, comprehensive research comparing the current implants (BIA400 and Ponto Wide) in terms of surgical outcomes has not been conducted, except for two small subanalyses in the studies of Lindeboom *et al*.^6^ and Van der Stee *et al*.^7^ Our study comprises a larger study population; extended follow-up and implant stability will be compared as well. The applicability of the Implant Stability Quotient (ISQ) is not yet fully known^8^ but may have a predictive value. Sennerby and Meredith^9^ established that, in dental implants, low or decreasing ISQ values are associated with increased risk for implant complications. In hearing implants, this relation has not yet been demonstrated.

The aim of this study is to investigate the differences in surgical outcomes between the BIA400 and Ponto Wide, thereby allowing for a more informed choice, based on factors beyond mere preference for the sound transducer. The primary outcome is focused on the relation between type of implant and ISQ, implant loss, soft tissue reaction and skin thickening. The secondary outcome is exploring if there is a relation between implant loss, soft tissue reaction and skin thickening on the one hand and the ISQ on the other hand.

## Materials and methods

### Patients

This single-centre, retrospective cohort study was conducted at the Deventer Hospital, a large general teaching hospital in the Netherlands. Between January 2013 and December 2023, a total of 220 BCDs were implanted in Deventer Hospital. This large patient cohort received a BCD due to conductive or mixed hearing loss or single-sided deafness, in combination with intolerance for, or lack of benefit from, a conventional hearing aid.

Patients with implant type BIA400 or Ponto Wide with at least two follow-up appointments were included. Children (< 18 years) were excluded, because the softer/thinner bone of their skull could influence the likelihood of complications. Patients who lost their implant due to surgical intervention or having undergone a change of abutment during follow-up, were included up to that point. Re-implants were considered as a new case.

### Peri-operative procedure

The indication for considering a BCD was determined by an ENT physician and an audiologist. This was followed by a period of testing various types of sound transducers. For this purpose, a head-band on which different types of sound transducers could be attached, was used. After several weeks of testing, patients expressed their preference and the surgery was scheduled. The length of abutment was depending on the patient’s skin thickness. The surgeries were performed by experienced implant surgeons (P.K., S.B.) using the linear incision technique with tissue preservation (LITT-P). Several studies suggest a preference for this technique due to significantly fewer adverse soft tissue reactions (Holgers Scale score ≥ 2).^6^^,^^7^ its less invasive nature and shorter operation time.^10^ During standard follow-up appointments at one week, three weeks, six months post-operatively and then annually or every other year, clinical outcomes such as implant stability, soft tissue reaction and skin thickening were evaluated.

### Outcome variables

Implant stability was measured objectively and non-invasively peri-operatively and during the follow-up appointments by using resonance frequency analysis (RFA).^11^ On both the BIA300/400 as well as the Ponto Wide abutments, Osstell® SmartPeg have been used at Deventer Hospital from 2013 onward. The SmartPeg provides two outcomes, ISQ-High and -Low, because the measurement is conducted in two perpendicular directions. In this study, only the ISQ-High is used.

In this study, complications refer to the most commonly reported tolerability problems in BCDs, that is, soft tissue reaction and skin thickening, as well as the less common but important complication, implant loss. Soft tissue reaction was objectified according to the Holgers score.^12^^,^^13^ A Holgers Scale score of 2 or greater was considered as an adverse soft tissue reaction because of the indication for (local) treatment. Skin thickening was categorised as either absent, present or present and requiring treatment. Treatment may include triamcinolone acetonide injection, change of abutment or surgical skin reduction. Implant loss was noted in the database as present or absent.

### Data collection

Findings and treatment were documented in the electronic patient record. In addition to the data previously mentioned, the following parameters were also recorded: age, gender, risk factors (smoking, cardiovascular risk factors, diabetes mellitus, mental retardation and body mass index [BMI]), indication for BCD, type of tissue at the bottom of the drilled hole, time until implant loss and months of follow-up.

### Statistical analysis

Statistical analysis was performed using SPSS version 28.0 (IBM, Armonk, USA), with a significance level of 5 per cent (*p* < 0.05). The baseline characteristics were compared using the chi-squared or Fisher’s exact test for categorical variables. The possible difference in numeric variables was determined by using the unpaired t-test in case of normally distributed data or Mann–Whitney *U*-test in case of non-normally distributed data. The Kolmogorov–Smirnov test was used to determine if there was a normal distribution.

For determining whether there was a difference between implant stability and type of implants, the ISQ-High measurements one year post-implantation was used, since osseointegration is expected to be completed after one year.^14^^,^^15^ Because of ISQ’s great dependence on abutment length,^16^ a subanalysis was made to show the difference per abutment length as well. Normally distributed ISQ data were analysed using the unpaired t-test. Not normally distributed ISQ data were analysed using the Mann–Whitney *U*-test for type of implant and Kruskal–Wallis for abutment length.

The Kaplan–Meier method was used in combination with log-rank or Breslow test, to assess differences between implant types and time until soft tissue reactions and skin thickening. In case of significant difference, Cox regression was performed to identify any confounding variables.

Decrease has been defined as a drop of one or more ISQ points relative to the expected ISQ. The expected ISQ, visualised in plots, has been predicted based on the baseline ISQ plus the median change over time. Stability indicates that the measured ISQ is equal to the expected ISQ at that point in time. An increase has been defined as a rise of one or more points relative to the expected ISQ based on the baseline ISQ plus the normal progression of ISQ as predicted. Descriptive statistics have been used due to the studies’ retrospective design and, therefore, variety in the number of follow-up visits.

For examining the relation between ISQ and implant loss, the last ISQ measurement taken before extrusion has been used.

## Results

### Baseline characteristics

**Figure 1.**
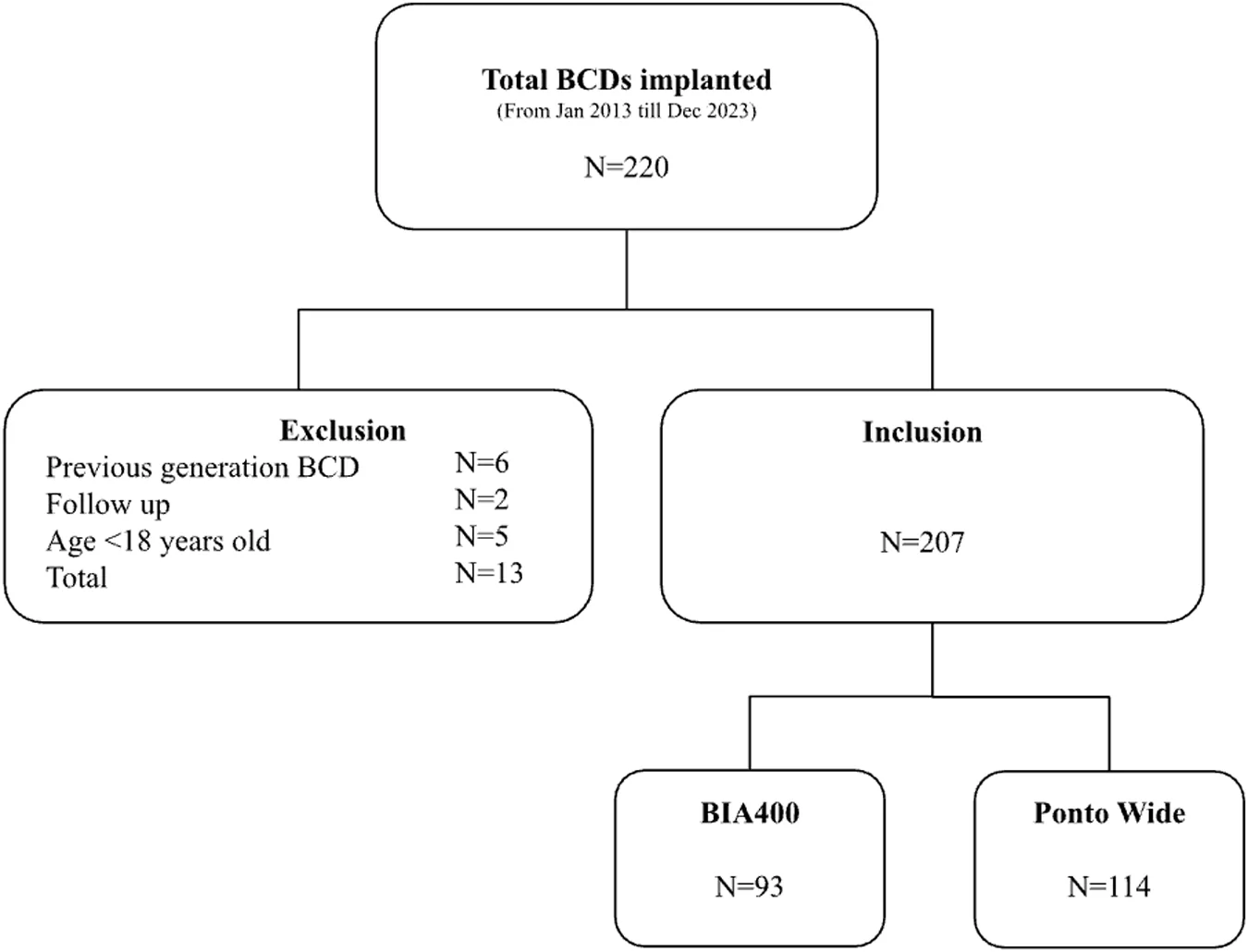
Inclusion flowchart.

A total of 220 BCDs were implanted between January 2013 and December 2023. After exclusion based on the criteria mentioned, 207 implants remained for analysis (see figure 1). Because of bilateral implantation and re-implantations, implants were distributed among 199 patients. Baseline characteristics are visualised in Table 1. Only abutment length appeared to be significantly different between the two groups.

**Table 1.** Baseline characteristics Table 1 long description.

Parameter	BIA400	Ponto wide	*p*-Value
Patient’s characteristics			
Total implants (%)	93 (44.9%)	114 (55.1%)	
Mean age at surgery (±SD)	59 (±15.2)	58 (±14.3)	0.898
Gender			0.531
- Male	40 (43.0%)	54 (47.4%)	
- Female	53 (57.0%)	60 (52.6%)	
Etiology of hearing loss			0.322
- Acquired conductive/mixed hearing loss	74 (79.6%)	84 (73.7%)	
- Single-sided deafness	19 (20.4%)	30 (26.3%)	
Risk factors (%)			
- Mean BMI (±SD)	28.0 (±4.8)	27.1 (±5.3)	0.061
- Smoking	21 (22.8%)	20 (18.0%)	0.396
- Diabetes mellitus	11(11.8%)	12 (10.5%)	0.767
- Cardiovascular comorbidity	44 (47.3%)	44 (38.6%)	0.207
- Mental retardation	3 (3.2%)	5 (4.4%)	0.733
Surgical characteristics			
Side			0.302
- Right	54 (58.1%)	58 (50.9%)	
- Left	39 (41.9%)	56 (49.1%)	
Surface drill hole			0.484
- Bone	76 (81.7%)	87 (76.3%)	
- Dura	9 (9.7%)	15 (13.2%)	
- Partial bone and dura	7 (7.5%)	12 (10.5%)	
- Vein	1 (1.1%)	0 (0.0%)	
Depth of drill hole			0.132
- 3 mm	1 (1.1%)	6 (5.3%)	
- 4 mm	92 (98.9%)	108 (94.7%)	
Abutment length			<0.001*
- 6.0 mm	3 (3.2%)		
- 8.0 mm	27 29.0%)		
- 9.0 mm		79 69.3%)	
- 10.0 mm	40 43.0%)		
- 12.0 mm	23 (24.7%)	35 (30.7%)	
**Follow-up**			
Mean follow-up in months (±SD)	44 (±32.8)	45 (±33.2)	0.720

* Statistically significantly different. BMI = body mass index; SD, standard deviation.

### Implant stability

The ISQ data from one year post-surgery were normally distributed without a significant difference (*p* = 0.882) between the mean ISQ being 60.2 (SD ±6.1) for the BIA400 and 60.4 (SD ±5.8) for the Ponto Wide. However, the ISQ value has great dependence on abutment length, which was confirmed (*p* < 0.001) in this study. The BIA400 and Ponto Wide share one common abutment length, namely the 12-mm abutment. To be more accurate about the difference in implant stability between the two types of implants, an extra analysis was conducted for only the 12-mm abutment. The ISQ values at one year post-surgery were normally distributed. No significant difference (*p* = 0.588) was found between the mean ISQ at one year between the BIA400 56.1 (SD ±4.5) and Ponto Wide 57.1 (SD ±4.1).

### Implant loss

In this cohort, one BIA400 (98.9 per cent implant survival) and three Ponto Wide (97.3 per cent implant survival) implants were lost. Three implants had a 12-mm abutment and one implant had a 9-mm abutment. One implant was lost after a period of pain and inflammation (BIA400), one implant after trauma (Ponto Wide 9 mm) and for two implants the reason for implant loss remained unclear (Ponto Wide). All the Ponto Wide implants were lost between six and 16 months after implantation. The BIA400 implant was lost 95 months after implantation.

### Soft tissue reaction

**Figure 2.**
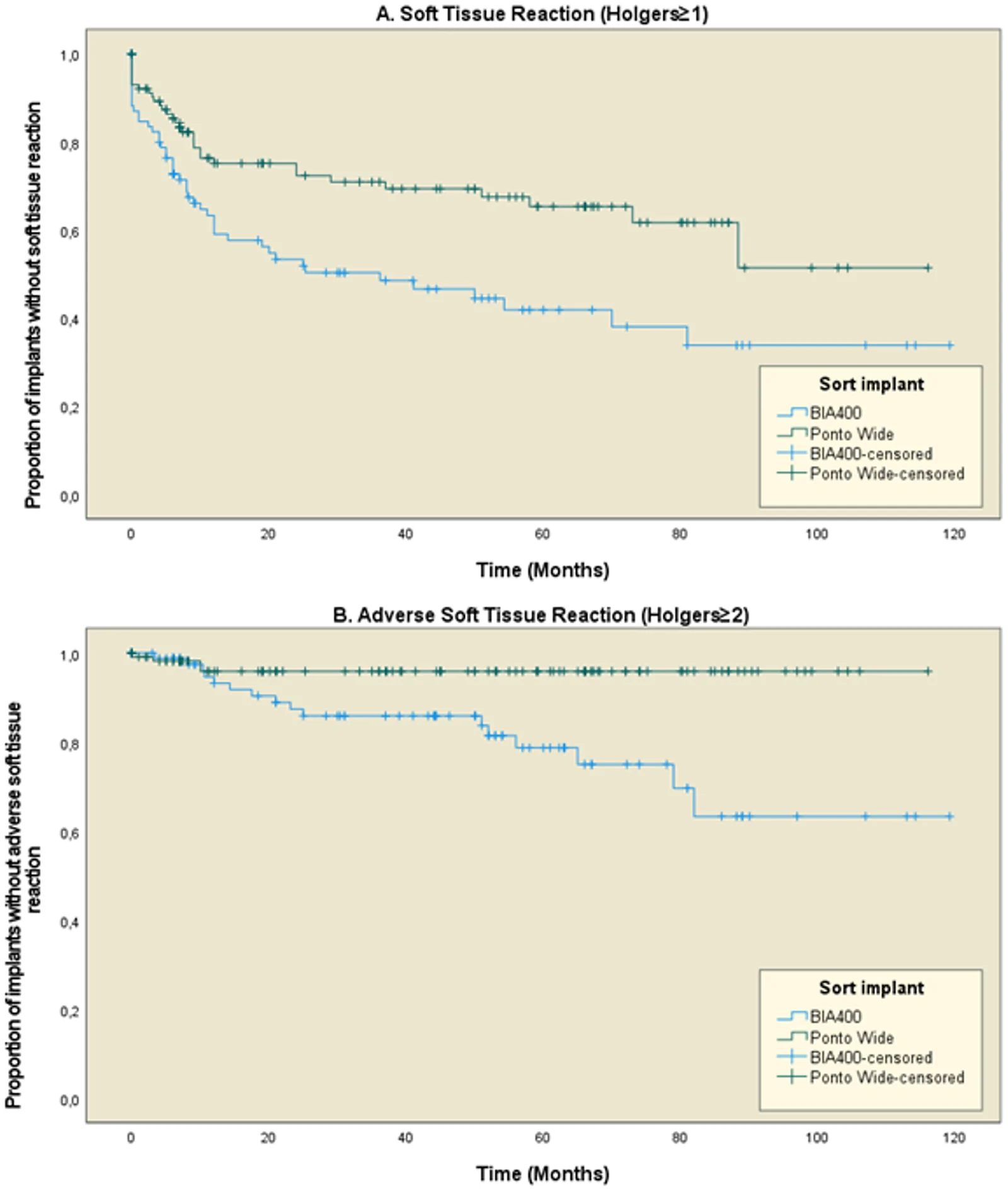
Kaplan–Meier survival curves showing time to soft tissue reaction. A. Soft tissue reaction Holgers ≥ 1 (*p* = 0.003). B. Adverse soft tissue reaction Holgers ≥ 2 (*p* = 0.018). Figure 2 long description.

In 46 BIA400 (49.5 per cent) and 33 Ponto Wide (28.9 per cent) implants, soft tissue reactions (Holgers ≥ 1) were seen during follow-up. Adverse soft tissue reactions (Holgers ≥ 2) showed up in 16 BIA400 implants (17.2 per cent) and four Ponto Wide (3.5 per cent). The likelihood of both soft tissue reaction as well as adverse soft tissue reaction, was significantly higher in the BIA400 group, as shown in Figure 2. A Cox regression analysis showed a significant influence of abutment length for either soft tissue reaction *(p* = 0.029) or adverse soft tissue reaction (*p* = 0.024). A total of seven implants showed a Holgers grade 3, of which two were Ponto Wide implants and five were BIA400 implants. All implants were treated with hydrocortisone/oxytetracycline/polymyxin B ointment and a healing cap, while also receiving systemic treatment with clindamycin or amoxicillin–clavulanic acid. None of these implants were lost.

### Skin thickening

**Figure 3.**
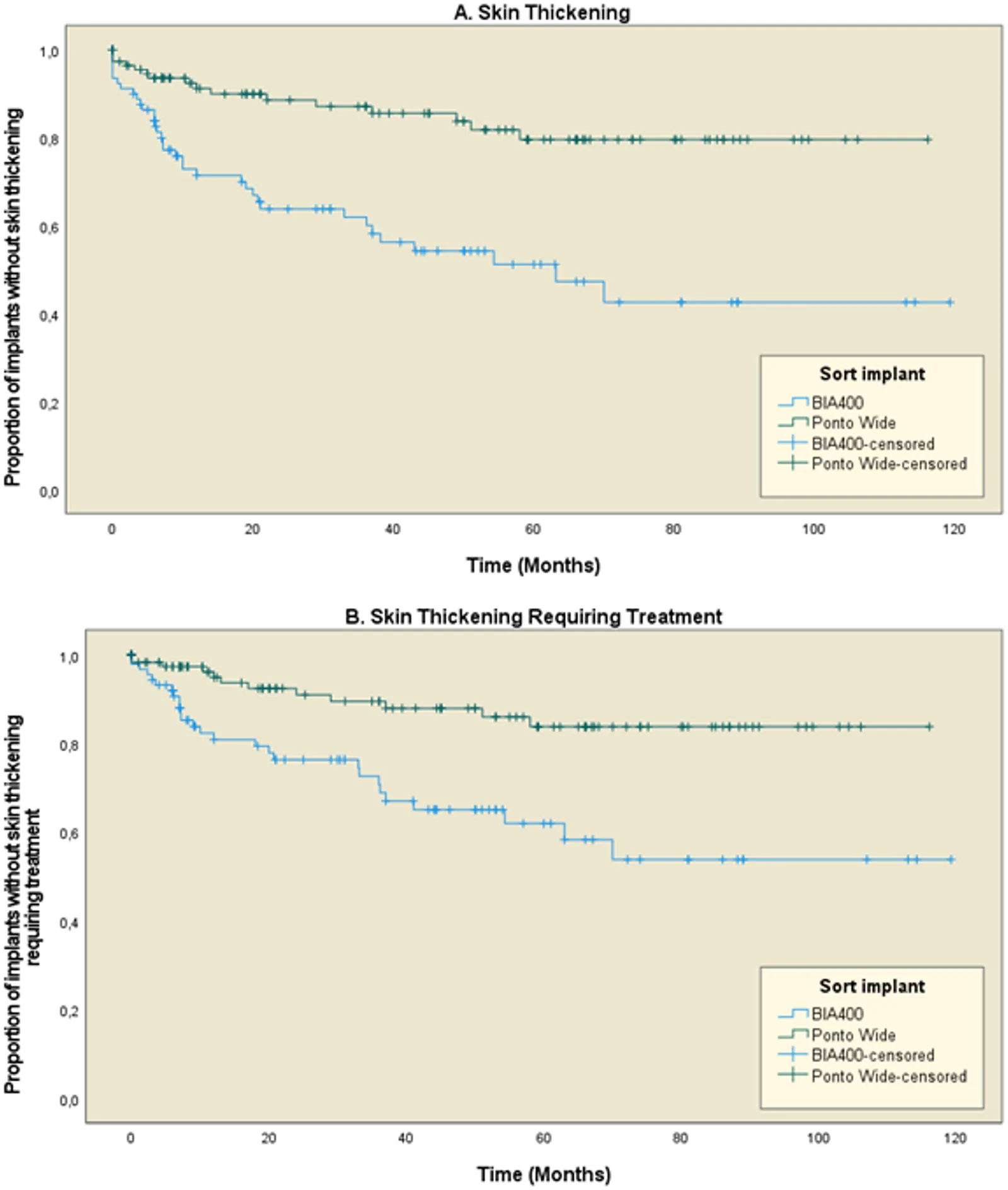
Kaplan–Meier survival curves showing time to skin thickening. A. Skin thickening (*p* < 0.001) B. Skin thickening requiring treatment (*p* < 0.001). Figure 3 long description.

Skin thickening was present in 36 BIA400 (38.7 per cent) and 16 Ponto Wide (14.0 per cent) implants. In 27 BIA400 (29.0 per cent) and 12 Ponto Wide (10.5 per cent) implants, skin thickening requiring treatment was seen during follow-up. Both were significantly more prevalent among patients with the BIA400 implants compared to those with the Ponto Wide implants, according to the Kaplan–Meier method (both *p* < 0.001), as shown in figure 3. Abutment length was found to be a significant covariable in both skin thickening (*p* < 0.001), and skin thickening requiring treatment (*p* = 0.001).

### Implant stability and complications

**Figure 4.**
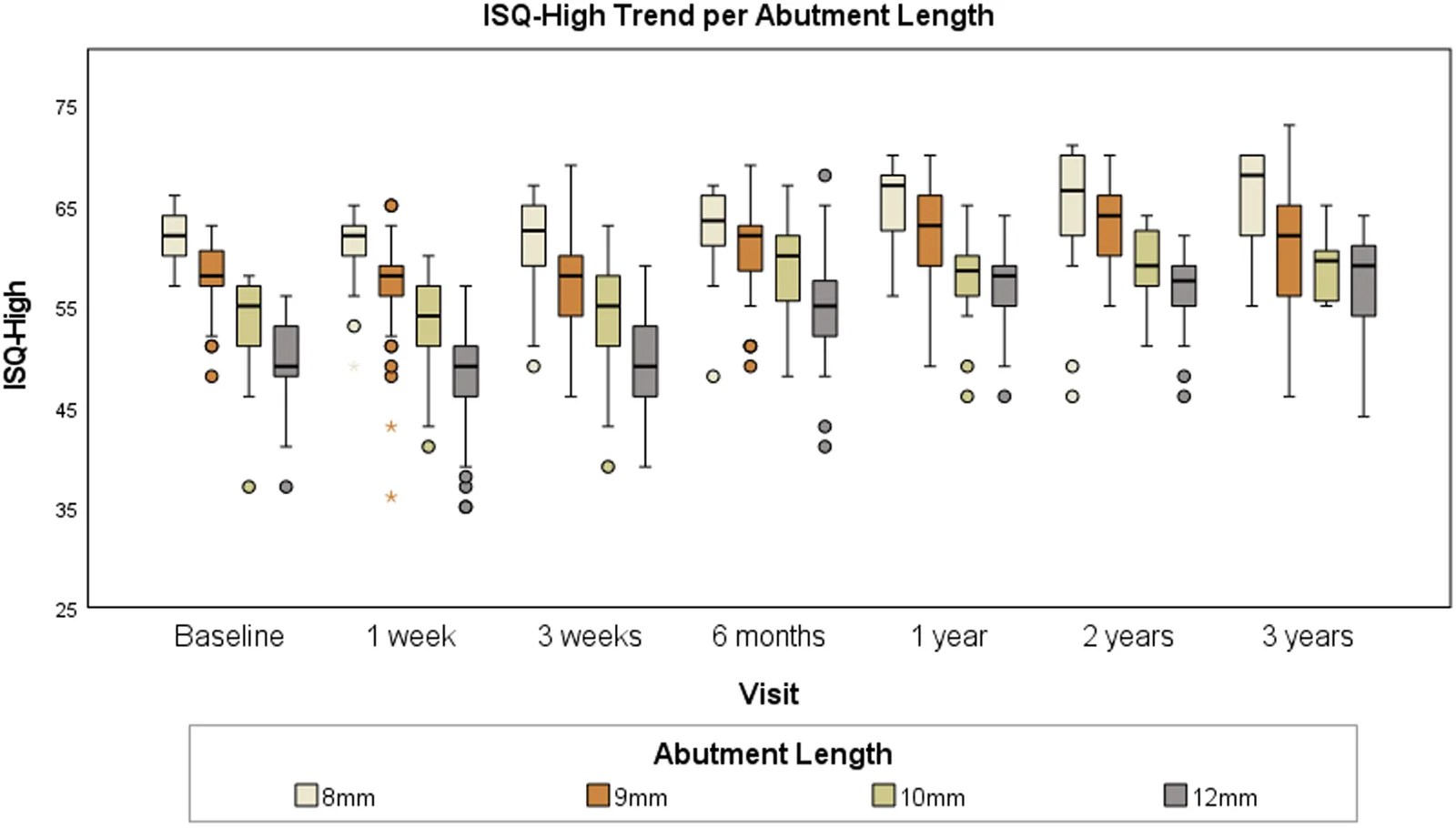
Boxplot of ISQ trend on date of surgery and standard follow-up visits, per abutment length. Figure 4 long description.

As previously demonstrated, ISQ values appeared to be strongly dependent on abutment length. Additionally, a significant amount of ISQ data was missing at each measuring point. Therefore, the ISQ trend was plotted for each abutment length as shown in Figure 4. The average follow-up period was 44 months, leading to the selection of a trend line up to year 3 post-implantation. There were only three implants with an abutment length of 6 mm, and due to the lack of ISQ data, a reliable ISQ trend line could not be established for these implants.

The figure shows that the ISQ typically is quite stable during the first three weeks, followed by a rapid increase in ISQ up to six months, after which the increase tapers off, reaching a plateau phase in the first year.

**Figure 5.**
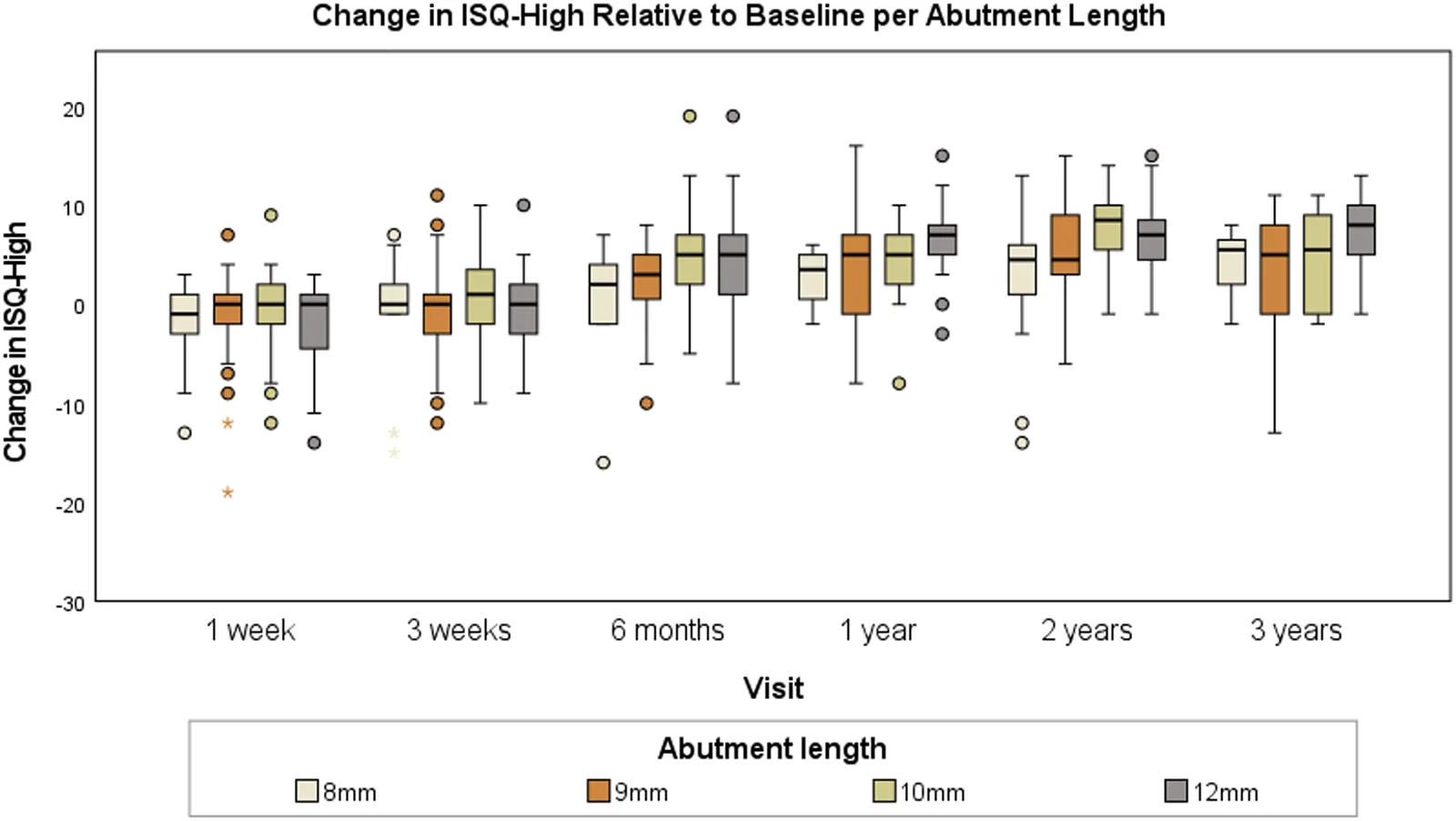
Boxplot of change in ISQ values relative to baseline on standard follow-up visits, per abutment length. Figure 5 long description.

The trend line was also plotted as a change per follow-up relative to the baseline, as shown in Figure 5. The median change in ISQ for all abutment lengths was unchanged relative to the baseline at week 1 and week 3, plus four points at six months and plus five points from one year onwards.

We attempted to analyse the possible relation between change in ISQ and soft tissue reaction or skin thickening, as outlined in the methodology. However, because no significant correlation was demonstrated and the database proved to be suboptimal for addressing this research question, we decided not to include these data.

Finally, the association between implant stability and implant loss was examined. Two implants, which were lost after trauma and a period of pain and inflammation, showed an eight-points decrease in ISQ during their last measurement taken before extrusion. Last ISQ values were measured three and 12 months before implant loss, respectively. The other two implants, for which the reason for extrusion was unclear, had an increase in ISQ of two or eight points during measurements taken one and six months before implant loss, respectively.

## Discussion

### Key results

The primary aim of this large retrospective cohort study was to compare two percutaneous BCDs. The BIA400 more frequently developed soft tissue reactions and adverse soft tissue reactions, as well as skin thickening and skin thickening requiring treatment compared to the Ponto Wide. Implant stability was not significantly different at one year post-operatively. Both implants had a very high survival rate, with only one lost implant (98.9 per cent) for the BIA400 and three lost implants (97.3 per cent) in the Ponto Wide group.

For the secondary outcome, the presence of a correlation between implant stability and complications was examined. The ISQ trend remained stable during the first two follow-up moments after implantation, after which the ISQ values increased. Between six months and its first year after implantation the plateau phase was reached, which was on average five points higher than the baseline ISQ. Our data analysis could not demonstrate a significant correlation between change in ISQ and soft tissue reaction or skin thickening, because our database proved to be suboptimal for conducting a sound statistical analysis. However, in individual patients, we did not notice clear changes in ISQ-value during those events. Furthermore, the number of extrusions was too small and lacking ISQ measurements shortly before (nearest was one month).

### Comparison with other studies

This study is the first study to primarily focus on the differences between the currently most used percutaneous BCDs: BIA400 and Ponto Wide. Nonetheless, in two previous retrospective studies a subanalysis was devoted comparing these two implants, using similar methods. Lindeboom *et al*.^6^ analysed a cohort size of 146 implants with a follow-up time between six months and seven years, and van der Stee *et al*.^7^ used a cohort of 58 implants with six months to three years follow-up. In terms of soft tissue reaction and skin thickening, results were consistent. The differences in tolerability between the two implants may be explained by the hydroxyapatite coating of the BIA400 abutment versus the titanium surface of the Ponto Wide.^4^^,^^5^ Another explanation could be the increased diameter of the BIA400 abutment in the skin, causing more friction compared to the Ponto Wide in which the diameter of the abutment increases above the skin. In contrast to this study, previous studies did not find a significant difference in terms of adverse soft tissue reaction^6^ and skin thickening requiring treatment.^7^ This can be explained by our longer follow-up period and the larger cohort size compared to the other studies.

The survival scores in this study were 98.9 per cent for the BIA400 and 97.3 per cent for the Ponto Wide. These results were in line with previous studies, which reported survival rates from 97.9 to 100 per cent for the BIA400^17^^,^^18^ and 97.9 to 100 per cent for the Ponto Wide.^15^^,^^19^^,^^20^ Our study observed that the three lost Ponto Wide implants became loose within a few months, while the BIA400 implant took several years before failing. These results are not consistent with those of Van Hoof *et al*.,^17^ where the BIA400 implant was lost within three months after implantation, or Reznitsky *et al*.,^20^ where the only lost implant failed a few years after implantation. This once again highlights that no conclusions can be drawn from the small number of losses.

In this study, an overall increase in ISQ-High has been observed. The plateau phase was reached between six months and one year and was on average five points above the baseline ISQ value. This could indicate that osseointegration was completed and the implant continues to stabilise after the initial tightening during surgery. This is in line with other studies.^14^^,^^15^^,^^19^^,^^21^^–^^23^ However, the progression leading to the plateau phase differed per study. Some studies^14^^,^^22^^,^^23^ found a decrease at one week, while others^19^^,^^21^ reported a stable increase from week one onwards. One study^15^ identified a peak at one week, followed by a slight decrease, and then a subsequent increase. There is no clear explanation for this discrepancy; it might be due to the practice in some places of tightening the abutments before measurement.

Even though Sennerby and Meredith^9^ established that in dental implants low or decreasing ISQ values point to an increased risk for implant complications, our retrospective study lacked sufficient data to statistically support this for BCDs in a sound manner.

### Strengths and limitations

The length of follow-up and the large cohort size are considered to be the main strengths of this study. Another asset of this study is that all surgeries were performed by the same two experienced surgeons in one clinical centre. Also, follow-up was well arranged with standard contact moments. Furthermore, the baseline characteristics of the two implants were very comparable, as only abutment length differed significantly.

The following limitations should be taken into consideration: First, the retrospective design results in a heterogeneous group. Moreover, to assess the difference in tolerability, the Kaplan–Meier curves were used. This method does not make the distinction whether certain implants experienced toleration problems once or repeatedly. Nevertheless, this analysis still indicates that BIA400 implants developed tolerability problems earlier and that more Ponto Wide implants never experienced toleration problems. Moreover, in various previous studies^6^^,^^7^^,^^24^ Kaplan–Meier method was utilised for soft tissue reaction and skin thickening as well, allowing comparative analysis. In addition, the low incidence of implant loss (one vs. three cases) limits the ability to perform meaningful statistical comparisons between the devices. These data are presented descriptively, consistent with best practice for rare events. Future studies with larger sample sizes are needed to evaluate potential differences in implant survival more robustly. Lastly, some patients experienced complaints and received medical advice and treatment through telephone consultation, so Holgers score and skin thickening could not be scored and RFA was not performed.

### Implications and future developments

A prospective study could possibly identify the exact ISQ threshold values, providing a more valid answer about if and which amount of change could predict complications. If the correlation is not demonstrated again, it should be questioned whether continued ISQ measurement is meaningful. However, if the correlation is confirmed, possible applications could include determining the moment for safe loading, recognising complete osseointegration, predicting complications before they become visible and therefore more treatable and objectifying the clinical outcomes of treatments.^9^ Finally, this study evaluated the differences in tolerability between the two implants without specified analysis for patient characteristics. Future research might determine the difference in these characteristics, making it possible to provide improved individual advice.

Obviously, it is crucial that the implant causes minimal complications and the patient is satisfied with the sound quality. As aforementioned, the Ponto Wide outperforms the BIA400 in terms of tolerance. Additionally, Kruyt *et al*.^25^ demonstrated that the costs for the BIA400 are higher compared to the Ponto Wide. Despite the clear advantages of the Ponto Wide over the BIA400, patients’ preference for sound transducer will still be an important factor in choosing the implant, while most complications are well manageable. Above all, it is important that the patient is properly informed about the differences in the complication rates.

What is already known on the subjects

- Two previous studies included small subanalyses suggesting that Ponto Wide implants may have better outcomes regarding soft tissue reactions and skin thickening compared to BIA400 implants
- High implant survival rates have been observed for both BIA400 and Ponto Wide devices, but direct comparisons were limited

What this study adds to our understanding

- It confirms that Ponto Wide implants result in fewer adverse soft tissue reactions and less skin thickening requiring treatment compared to BIA400 implants
- Both implants demonstrated high survival rates: 98.9 per cent for BIA400 (one lost implant) and 97.3 per cent for Ponto Wide (three lost implants)
- Implant stability was comparable between the two groups, even when considering only the 12-mm abutments
- ISQ trends showed a consistent increase starting three weeks post-implantation, reaching a plateau phase between six months and its first year

## Conclusion

This study demonstrates that both implants have excellent implant survival rates and similar implant stability for the total implant group, but favourable outcomes in terms of tolerability for the Ponto Wide. Future research should focus per implant on determining whether the difference in patient characteristics influences the risk of complications, thereby allowing to provide individual advice. Furthermore, ISQ trends showed an increase after three weeks post-implantation reaching its plateau phase between six months and its first year. Lastly, it was not possible to conduct a statistically sound analysis of the relationship between complications and a decrease in ISQ-High, despite the fact that, in individual patients, we did not notice decreased ISQ values at the moment of skin reactions or skin thickening. Future prospective research should focus on statistically clarifying the relation between implant stability and complications.

## Statements of declarations

No funds, grants or other support was received.

## Figure 2 Long description

The image A showing a line graph titled A. Soft Tissue Reaction (Holgers greater than or equal to 1). The horizontal axis label is Time (Months), with values from 0 to 120. The vertical axis label is Proportion of implants without soft tissue reaction, with values from 0.0 to 1.0. A legend titled Sort implant lists BIA400, Ponto Wide, BIA400-censored and Ponto Wide-censored. Two step lines are plotted with plus markers along the lines. The BIA400 line starts near 1.0 at 0 months, drops to about 0.75 by about 10 months, stays near 0.75 to about 20 months, then declines in steps to about 0.65 by about 60 months, stays near 0.65 to about 80 months, then drops to about 0.50 near about 85 months and remains near 0.50 through 120 months. The Ponto Wide line starts near 1.0 at 0 months, drops to about 0.60 by about 10 months, declines to about 0.50 by about 20 months, then declines in steps to about 0.40 by about 60 months, drops to about 0.35 near about 75 to 80 months and remains near 0.35 through 120 months. The image B showing a line graph titled B. Adverse Soft Tissue Reaction (Holgers greater than or equal to 2). The horizontal axis label is Time (Months), with values from 0 to 120. The vertical axis label is Proportion of implants without adverse soft tissue reaction, with values from 0.0 to 1.0. A legend titled Sort implant lists BIA400, Ponto Wide, BIA400-censored and Ponto Wide-censored. Two step lines are plotted with plus markers along the lines. The BIA400 line starts near 1.0 at 0 months and stays near 1.0 through 120 months with small step decreases, ending near about 0.95 to 1.0 at 120 months. The Ponto Wide line starts near 1.0 at 0 months, declines to about 0.90 by about 20 months, stays near about 0.90 to about 50 months, then declines in steps to about 0.80 by about 60 months, drops to about 0.65 near about 80 months and remains near 0.65 through 120 months.

Navigate back to Figure 2.

## Figure 3 Long description

The image contains two Kaplan–Meier survival curves comparing skin thickening outcomes over time for different implant types. The horizontal axis represents time in months, ranging from 0 to 120. The vertical axis represents the proportion of implants without skin thickening, ranging from 0.0 to 1.0. In the first graph, labeled ′A. Skin Thickening′, the BIA400 line shows a higher proportion of implants without skin thickening compared to the Ponto Wide line. The curves begin to diverge around 12 months, with BIA400 maintaining a higher proportion throughout. At 24 months, BIA400 is approximately at 0.9, while Ponto Wide is around 0.7. By 60 months, BIA400 remains above 0.8, whereas Ponto Wide drops below 0.6. At 120 months, BIA400 is still above 0.7, while Ponto Wide is near 0.5. Censored data points are marked with plus symbols. In the second graph, labeled ′B. Skin Thickening Requiring Treatment′, similar trends are observed. BIA400 maintains a higher proportion of implants without requiring treatment compared to Ponto Wide. At 12 months, BIA400 is near 0.95, while Ponto Wide is around 0.85. By 24 months, BIA400 is approximately 0.9 and Ponto Wide is about 0.75. At 60 months, BIA400 is above 0.85, while Ponto Wide is below 0.7. At 120 months, BIA400 remains above 0.8 and Ponto Wide is near 0.6. Censored data points are similarly marked. The graphs illustrate that BIA400 implants generally maintain a higher proportion without skin thickening and without requiring treatment over time compared to Ponto Wide implants. The lines are visually distinguished by different styles and markers, with censored data indicated by plus symbols.

Navigate back to Figure 3.

## Figure 4 Long description

The box plot illustrates the ISQ-High trend per abutment length across various visits: Baseline, 1 week, 3 weeks, 6 months, 1 year, 2 years and 3 years. The vertical axis represents ISQ-High values ranging from 25 to 75, with no specific units indicated. Each visit includes box plots for abutment lengths of 8mm, 9mm, 10mm and 12mm. The median, lower quartile and upper quartile are shown, with whiskers indicating variability. Outliers are marked outside the whiskers. Overall, the 12mm abutment length tends to have higher median ISQ-High values across visits. Variability is generally consistent, with some narrowing observed at 3 years. Notable outliers are present at several visits, particularly for the 8mm and 9mm lengths. The plot reveals trends in ISQ-High values over time, with some lengths showing slight increases in median values.

Navigate back to Figure 4.

## Figure 5 Long description

Change in ISQ-High Relative to Baseline per Abutment Length. The horizontal axis label is Visit. The categories are 1 week, 3 weeks, 6 months, 1 year, 2 years, 3 years. The vertical axis label is Change in ISQ-High. The vertical axis range is minus 30 to 20, with tick labels at minus 30, minus 20, minus 10, 0, 10, 20. Legend label: Abutment length. Legend categories: 8 millimeter, 9 millimeter, 10 millimeter, 12 millimeter. Each visit category contains four box-and-whisker plots, one for each abutment length. Each box shows the interquartile range with a median line. Whiskers extend above and below the box. Individual points are plotted outside the whiskers. Across the visits, the median lines for the boxplots are positioned around the 0 tick to the 10 tick. Several boxplots at later visits have median lines positioned higher than those at earlier visits. Outlier points are visible above and below multiple boxplots. The highest visible outlier points are near the 20 tick. The lowest visible outlier points are near the minus 30 tick. The plotted values extend within the displayed vertical axis range from minus 30 to 20.

Navigate back to Figure 5.

## Table 1 Long description

The table compares baseline patient and surgical characteristics for implants in two groups, BIA400 (93 implants) and Ponto Wide (114 implants), with p-values for between-group differences. Mean age at surgery was similar at about 59 years in BIA400 and 58 years in Ponto Wide, and gender distribution was comparable (about 43 percent male in BIA400 versus 47 percent in Ponto Wide). Etiology was also similar, with acquired conductive or mixed hearing loss making up roughly three quarters to four fifths of cases and single-sided deafness about one fifth to one quarter. Risk factors were broadly comparable: mean body mass index was about 28.0 versus 27.1, smoking about 23 percent versus 18 percent, diabetes about 12 percent versus 11 percent, cardiovascular comorbidity about 47 percent versus 39 percent, and mental retardation about 3 to 4 percent. Surgical characteristics showed no clear differences in side (right about 58 percent versus 51 percent) or drill-hole surface categories, and most drill holes were 4 mm deep in both groups. Abutment length was the only characteristic reported as clearly different between groups, but several abutment-length entries appear inconsistently formatted or incomplete, so the exact distribution should be interpreted with caution. Mean follow-up was similar at about 44 to 45 months.

Navigate back to Table 1.
